# A case of hand-foot syndrome with olaparib

**DOI:** 10.11604/pamj.2022.43.216.38384

**Published:** 2022-12-30

**Authors:** Rafael Everton Assunção Ribeiro da Costa, Rodrigo José de Vasconcelos Valença

**Affiliations:** 1 Health Science Center, State University of Piauí, Teresina (PI), Brazil

**Keywords:** Hand-foot syndrome, hysterectomy, vemurafenib

## Image in medicine

Hand-foot syndrome or palmar-plantar erythrodysesthesia is an adverse cutaneous reaction to chemotherapy, mainly associated with capecitabine, doxorubicin, sorafenib, sunitinib, and vemurafenib. Skin reaction is characterized by the abrupt onset of dysesthesia, erythema, swelling, and fine desquamation of the hands and feet. Treatment is nonspecific and early diagnosis causes a significant improvement in the quality of life of oncology patients. We describe a case of a 48-year-old white female patient, diagnosed with ovarian cancer (stage III) in 2018. The patient had been previously managed with an oncologic hysterectomy, followed by adjuvant chemotherapy with 6 cycles of a carboplatin and paclitaxel regimen. In November 2019, she experienced peritoneal tumor recurrence and was retreated with carboplatin and paclitaxel chemotherapy, showing radiological response and decreased CA-125 levels. Maintenance treatment with olaparib was initiated in April 2020, and 3 months afterward, she developed pain and irritation in her hands and feet. On clinical examination, erythema was identified by dryness and desquamation of the palms of the hands and soles of the feet consistent with palmar-plantar erythrodysesthesia. It was determined that olaparib treatment would not be interrupted. Treatment with thermal water was performed, followed by moisturizers and medium-potency topical corticosteroids (mometasone furoate 0.1%). The patient died of ovarian cancer progression in December 2021. Family members gave consent for the publication and signed the informed consent form (ICF).

**Figure 1:**
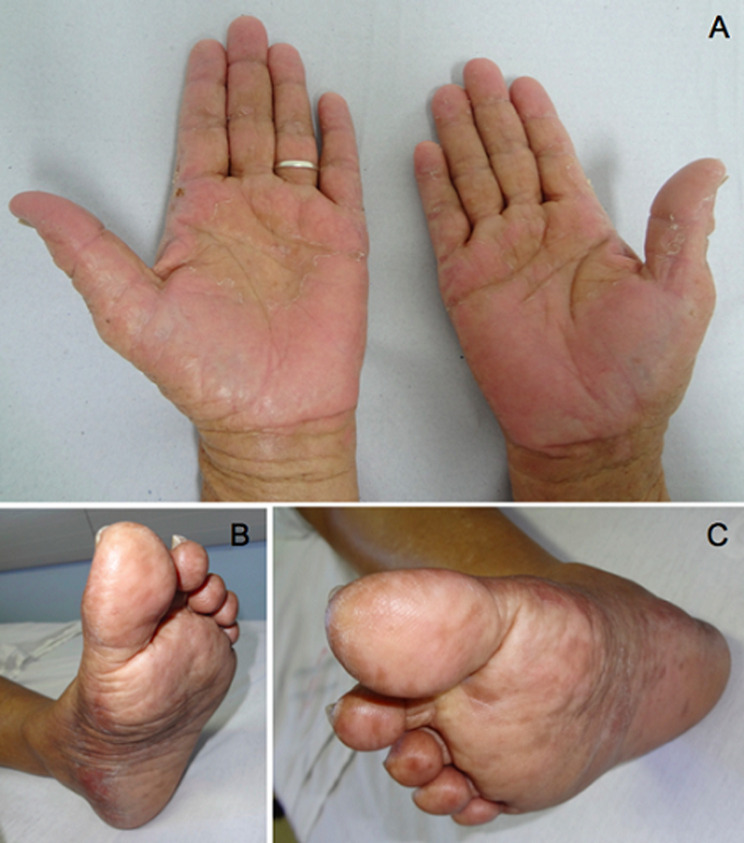
hand-foot syndrome with olaparib showing erythema with dryness and desquamation; A) palms of the hands; B, C) soles of the feet

